# A Vision for the Future of Neutron Scattering and Muon Spectroscopy in the 2050s

**DOI:** 10.1021/acsphyschemau.4c00026

**Published:** 2024-07-10

**Authors:** Stewart F. Parker, Peter J. Baker, Robert McGreevy

**Affiliations:** ISIS Neutron and Muon Source, STFC Rutherford Appleton Laboratory, Chilton, Didcot OX11 0QX, U.K.

**Keywords:** neutron scattering, muon spectroscopy, spallation source, reactor source, accelerator

## Abstract

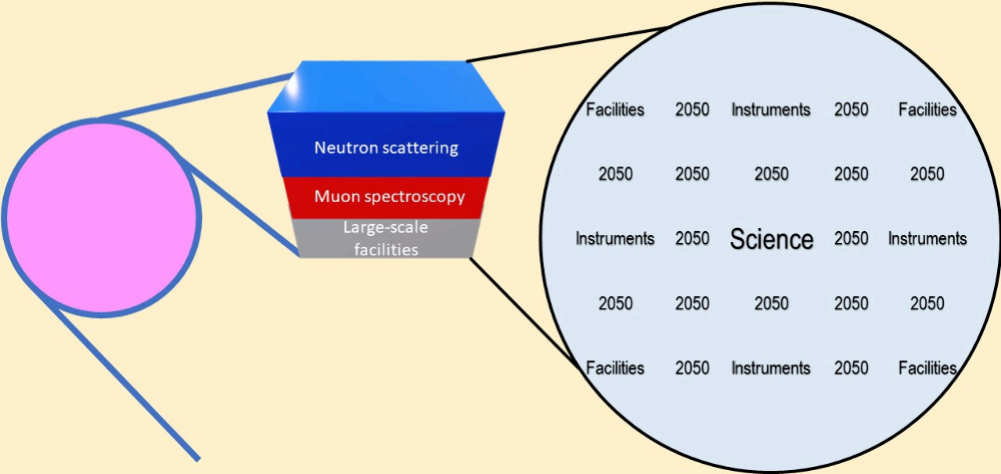

Neutron scattering and muon spectroscopy are techniques that use subatomic particles to understand materials across a wide range of energy (μeV to tens of eV), length (Å to cm) and time (attosecond to hour) scales. The methods are widely used to study condensed phase materials in areas that span physics, chemistry, biology, engineering and cultural heritage. In this Perspective we consider three questions: (i) will neutron scattering and muon spectroscopy still be needed in the 2050s? (ii) What might the technology to produce neutron and muon beams look like in the 2050s? (iii) What will be the applications in the 2050s? Overall, the neutron/muon ecosystem in the 2050s will have less capacity than now, but greater capability because of the somewhat higher power sources, better instrumentation and data analysis.

## 1 Introduction

**Figure 1:**
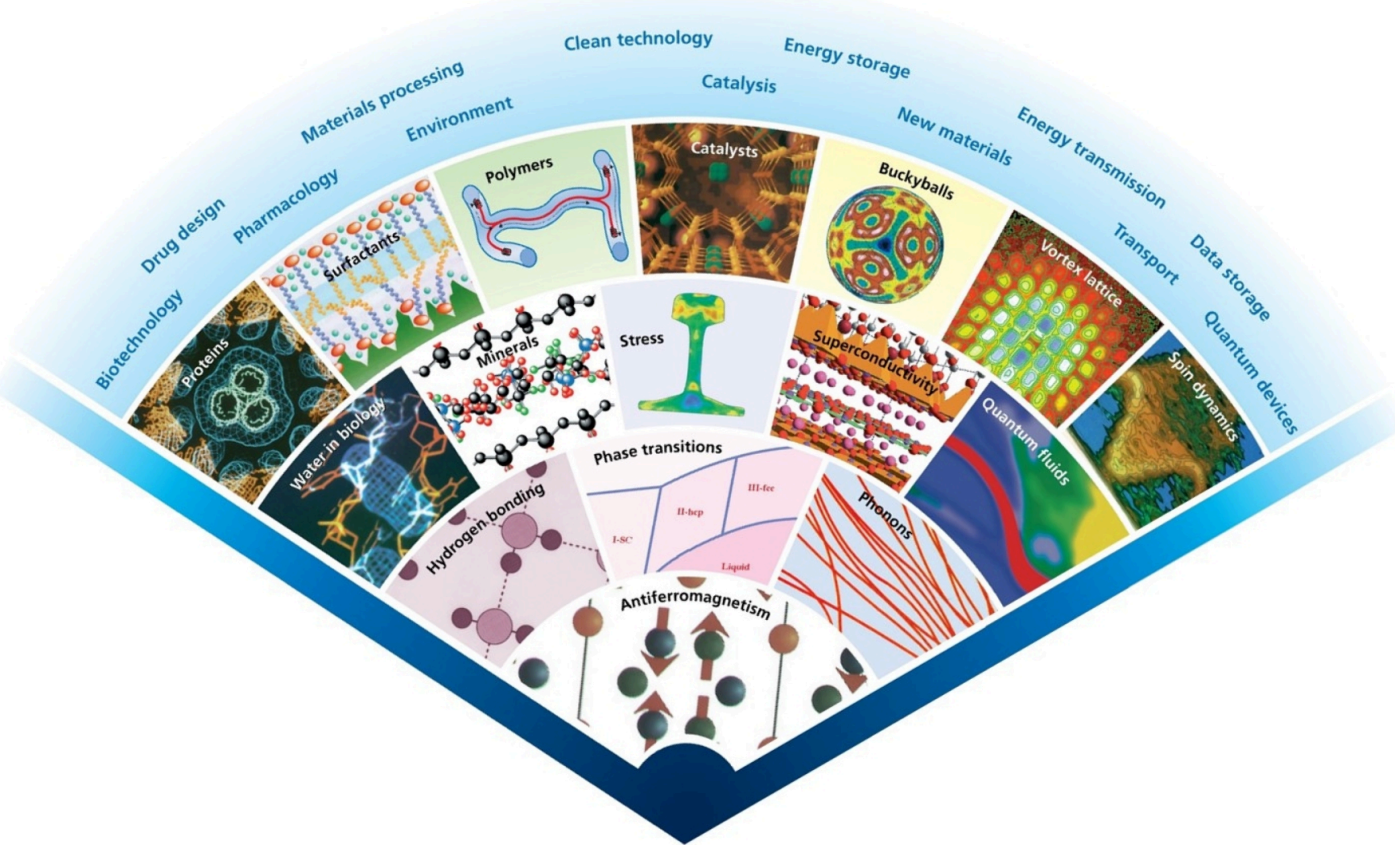
Picture shows how neutron scattering has evolved from its start in the mid-1950s (the center of the fan) to the present day (its outer edges). As the fan is traversed from right-to-left the applications change from condensed matter physics to chemistry to engineering to geology to biology, demonstrating how neutron science has moved far beyond its origins in physics to encompass many areas of contemporary science. Image created by Prof Bill David (University of Oxford and ISIS Neutron and Muon Facility).

Neutron scattering^1^ and muon spectroscopy^2^ are techniques that use subatomic particles to understand materials across a wide range of energy (μeV to tens of eV), length (Å to cm) and time (attosecond to hour) scales. The methods are widely used to study condensed phase materials in areas that span physics, chemistry, biology, engineering and cultural heritage.^3−6^ The range and variety of the applications are illustrated in Figure 1.

The origins of neutron scattering and muon spectroscopy lie in the 1930s with the discovery of the respective particles.^7,8^ However, it was not until the 1950s with the development of fission reactors and accelerator-based sources that provided intense beams, that the techniques became mainstream methods in condensed matter research. As of 2024, there are four major neutron scattering centers: the ISIS Neutron and Muon Facility (ISIS, Chilton, UK), the Institut Laue Langevin (ILL, Grenoble, France), the Spallation Neutron Source (SNS, Oak Ridge, USA) and the Japan Proton Accelerator Research Complex (J-PARC, Tokai, Japan) with a fifth, the European Spallation Source (ESS, Lund, Sweden), under construction. In addition, there are approximately 25 other sources (mostly low-power reactors) across the globe,^9^Table 1 gives a list of these. Spallation sources and reactor sources are complementary because of the different energy distribution they produce. Typically, reactors are optimized for the production of low energy neutrons (<100 meV), while spallation sources produce a much wider energy band (extending to 10s of eV) but with a lower time integrated flux. This disadvantage will be overcome by the ESS, which at full power (5 MW), is designed to match the total flux of the best reactor (the ILL).

**Table 1:** Neutron and Muon Facilities

Facility	Name, country, and Web site
BARC	Bhabha Atomic Research Centre (India) Research Reactors in BARC:Bhabha Atomic Research Centre(BARC), Department of Atomic Energy, Government of India
BNC	Budapest Neutron Centre (Hungary) https://www.bnc.hu/
CARR	China Advanced Research Reactor (China) http://www.ciae.ac.cn/zh401en/science_technology/cefr/index.html
CiADS	China initiative Accelerator Driven System (China) https://english.imp.cas.cn/research/facilities/CIADS/
CMRR	China Mianyang Research Reactor (China) http://english.ihep.cas.cn/ls/cnss/zzsszz/201406/t20140620_123024.html
CSNS	Chinese Spallation Neutron Source (China) http://csns.ihep.ac.cn/
ESS	European Spallation Source (Sweden) https://europeanspallationsource.se/
GEMS	German Engineering Materials Science Centre(Germany) https://www.hereon.de/central_units/gems/index.php.en
HANARO	High Flux Advanced Neutron Application Reactor (South Korea) http://hanaro.kaeri.re.kr/hanaroe
HFIR	High Flux Isotope Reactor (USA) https://neutrons.ornl.gov/hfir
IFE	Institute for Energy Technology (Norway) www.ife.no/
ILL	Institut Laue Langevin (France) https://www.ill.eu/about-the-ill
JINR	Joint Institute for Nuclear Research (Russia) https://flnp.jinr.int/en-us/
ISIS	ISIS Neutron and Muon Source (UK) https://www.isis.stfc.ac.uk/Pages/home.aspx
JCNS	Jülich Centre for Neutron Science (Germany) www.fz-juelich.de/jcns/
J-PARC	Japan Proton Accelerator Research Complex (Japan) https://j-parc.jp/researcher/index-e.html
JRR-3M	Japan Research Reactor (Japan) Research reactors and Accelerators/Japan Atomic Energy Agency/Nuclear Science Research Institute (jaea.go.jp)
LANSCE	Los Alamos Neutron Science Centre (USA) lansce.lanl.gov/
LLB	Laboratoire Léon Brillouin (France) www.llb.cea.fr/
MARIA	National Centre for Nuclear Research (Poland) https://www.ncbj.gov.pl/en
MLZ	Heinz Maier-Leibnitz Zentrum (Germany) http://www.mlz-garching.de/
MURR	University of Missouri Research Reactor Center (USA www.murr.missouri.edu/
NCNR	National Institute of Standards and Technology (NIST) Center for Neutron Research (USA) www.ncnr.nist.gov/
NPL	Nuclear Physics Laboratory (Czech Republic) https://www.ujf.cas.cz/en/
OPAL	Open Pool Australian Lightwater reactor(Australia) https://www.ansto.gov.au/facilities/opal-multi-purpose-reactor
ORNL	Oak Ridge National Laboratory (USA) https://neutrons.ornl.gov/
PNPI or IPPN	B.P. Konstantinov St. Petersburg Nuclear Physics Institute (Russia) https://www.nti.org/education-center/facilities/konstantinov-st-petersburg-nuclear-physics-institute-ippn-or-pnpi/
RA-10	Laboratorio Argentino de Haces de Neutrones (Argentina) https://www.lahn.cnea.gov.ar
RAON	Rare isotope Accelerator complex for Online experiments (South Korea) https://risp.ibs.re.kr/html/risp_en/
RID	Reactor Institute Delft (The Netherlands) https://www.tudelft.nl/en/faculty-of-applied-sciences/business/facilities/tu-delft-reactor-institute
RMB	Brazilian Multipurpose Reactor (Brazil)
SNS	Spallation Neutron Source (USA) https://neutrons.ornl.gov/sns
SINQ	Swiss Spallation Neutron Source (Switzerland) www.psi.ch/sinq
SμS	Swiss Muon Source (Switzerland) https://www.psi.ch/en/smus
TRIUMF	TRIUMF (Canada) https://www.triumf.ca/

There are four major muon spectroscopy centers. As muon production requires the same infrastructure as needed for spallation neutron sources, two of these are part of ISIS and J-PARC. The two other sources are the Swiss Muon Source (SμS, Villigen, Switzerland) and TRIUMF (Vancouver, Canada).

Neutron scattering and muon spectroscopy are flourishing fields as evidenced by the breadth of applications shown in Figure 1 and the ∼2500 publications per year. However, it is reasonable to ask if this will continue to be the case. In this Perspective, we will attempt to answer three questions:

- Will neutron scattering and muon spectroscopy still be needed in the 2050s?
- What might the technology to produce neutron and muon beams look like in the 2050s?
- What will be the applications in the 2050s?

## 2 Will Neutron Scattering and Muon Spectroscopy Still Be Needed in the 2050s?

To a large degree this question is rhetorical, because if the answer is “no”, then the second and third questions are irrelevant! We will attempt to demonstrate why we believe the answer is actually “yes” by considering the current uses for the techniques and whether there are realistic alternatives.

The usefulness of neutrons and muons derive from their fundamental properties and the key ones are listed in Table 2.

**Table 2:** Neutron and Muon Properties

Property	Neutron	Muon
Mass/amu	1.009	0.113
Charge/e	0	±1
Magnetic moment/μ_N_	–1.913	±8.89
Spin/ℏ	1/2	1/2
Lifetime/s	879	2.197 × 10^–6^

We will first consider the uses of neutron scattering. Neutrons are uncharged, so the scattering is largely from the atomic nuclei, mediated by the strong force. (Neutrons do possess a magnetic moment, so are also scattered by unpaired electrons, which has led to the applications of neutrons for the study of magnetism). As the diameter of an atomic nucleus is only 10^–5^ that of the atom, it follows that most of matter is empty space to a neutron and consequently they are highly penetrating. Thus 1 cm of steel will transmit 33% of the incident neutrons. This means that neutron scattering is dominated by the bulk of the material.

**Figure 2:**
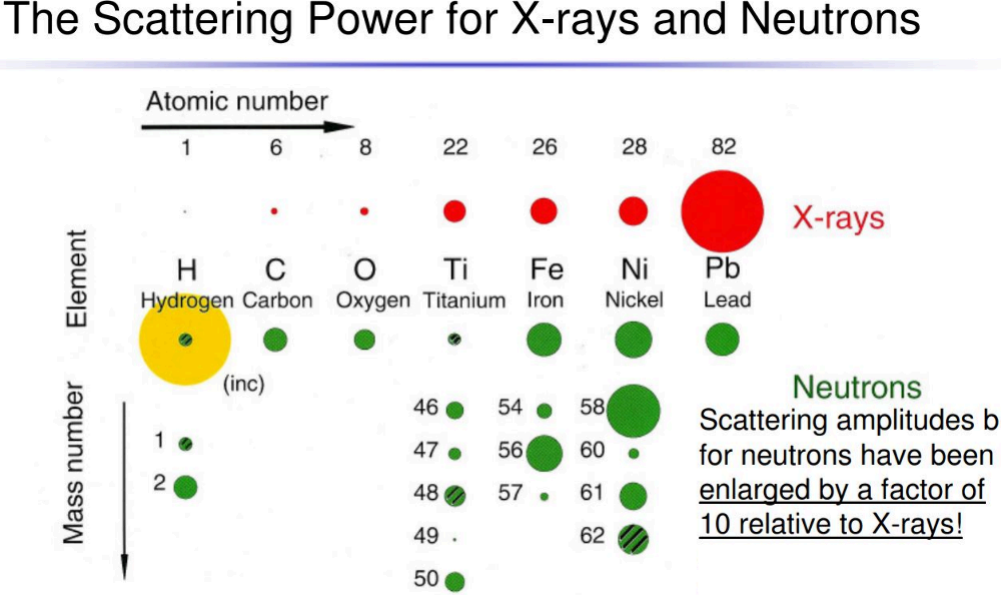
Comparison of neutron and X-ray scattering cross sections. Note that the neutron cross sections are both element and isotope dependent. (Isotopes that are cross-hatched have a negative scattering length i.e. the neutron undergoes a 180° phase shift on scattering). Reproduced with permission from ref [^13^]. Copyright 2007 International Centre for Theoretical Physics (ICTP).

This has enabled applications as diverse as neutron imaging of cultural heritage artifacts, strain mapping of engineering components and measurement of phonon dispersion relations. For all of these examples (and many others), the major “competitor” is X-rays, particularly synchrotron generated X-rays. Even a laboratory X-ray source is significantly brighter than a neutron source, thus applications where sensitivity is the prerequisite will eventually lose out to X-ray sources. An example is high pressure research. For the highest pressures, diamond anvil cells are used. At synchrotron sources pressures as high as 900 GPa^10^ have been achieved, while the best to date at a neutron source is 100 GPa.^11^ Hence, it would seem that high pressure research at neutron sources is pointless. This is not the case because of the fundamental difference between how X-rays and neutrons interact with matter: X-rays are scattered by electrons, neutrons by nuclei. Thus neutrons “see” light elements even in the presence of heavy atoms, see Figure 2, so, for instance, the structures of the high pressure phases of ice have largely been determined by neutron scattering, as X-rays will only provide the oxygen positions.^12^ As the hydrogen bonding is the determining factor in the structure, knowledge of the hydrogen positions is crucial to understanding the materials.

The nuclear scattering of neutrons has other consequences. As a nucleus is essentially a point source, there is no form factor for neutron diffraction, which results in high precision for structural studies, especially those involving hydrogen. The neutron scattering cross section is both element and isotope dependent, Figure 2, so by manipulating the isotopic composition, it is possible to gain contrast for neighboring elements that have very similar X-ray scattering power. Thus Mn, Fe, Co, Ni and Cu have neutron total scattering cross sections of 2.15, 11.62, 5.6, 18.5, and 8.03 barn (1 barn = 10^–28^ m^2^) respectively, whereas the X-ray cross sections change by less than 10% from Mn to Cu.

The most striking example of the isotopic dependence of the cross section is that of hydrogen and deuterium. These have coherent cross sections of 1.76 (H) and 5.592 barn (D) and incoherent cross sections of 80.27 (H) and 2.05 barn (D). This is exploited in small angle neutron scattering (SANS), neutron reflectometry and neutron total scattering to increase the information content (because the composition is known, the isotopic variant provides an additional observable without increasing the number of unknowns). Hydrogen and deuterium have negative and positive scattering lengths respectively (a negative scattering length means that the neutron undergoes a 180° phase shift on scattering), thus a mixture of 1.61 H_2_O: 1 D_2_O (mass ratio) will have zero coherent scattering length, thus making the solvent invisible to neutrons. Similarly, an alloy of the compositionTi_0.676_ Zr_0.324_, also has zero coherent scattering length, meaning that sample cans can be invisible. For neutron vibrational spectroscopy (inelastic neutron scattering, INS) and quasielastic neutron scattering (QENS), for hydrogenous materials selective deuteration is a means to mask part of the system.

At most neutron scattering centers, studies of magnetism and related phenomena account for 30–50% of beam time. This underlines the crucial role of neutrons in this area. The interaction is between the magnetic moment of the neutron and unpaired electrons in the material. Both elastic scattering^4,14^ (which provides crystallographic and magnetic structure simultaneously) and inelastic scattering,^4^ (which informs about the energy scales present and gives information on spin dynamics *e.g*. spin waves or magnons, spin fluctuations, crystal-field excitations) are both widely used. However, magnetic X-ray diffraction at synchrotrons^15^ has evolved very rapidly and for thin film or very small samples is the technique of choice and is likely to be an area where X-rays will eventually supersede the use of neutrons. Resonant inelastic X-ray scattering (RIXS),^16^ is becoming competitive with inelastic neutron scattering to study magnetic excitations,^17^ but cannot match the resolution in the ∼0–0.1 eV range where neutrons excel. It also complements some muon measurements today and might replace some use of muons in future but is unlikely to match muons’ particular sensitivity to smaller magnetic moments Thus neutrons and muons are still likely to be used, and needed, to study magnetism in the 2050s.

One area where there are no competing techniques is the study of diffusion in materials at the atomic scale by QENS^18^ and muon spin relaxation.^19^ This uniquely provides diffusion constants and activation energies at the atomic scale, with QENS also providing information on the geometry of motion. This is used in applications as diverse as catalysis (to measure how atoms/molecules move over/through a catalyst), soft matter (to study segmental motion in biomolecules and polymers) and materials science (to study movement of atoms *e.g*. H, Li, O, Na through materials that include proton or oxide conductors, batteries and metals).

One key feature of neutron scattering and muon spectroscopy is that both are nondestructive, in contrast to X-ray scattering. This is of particular significance for protein crystallography, where crystals are typically measured at 100 K by X-rays to minimize the radiation damage. Neutrons allow protein structures to be determined at physiological temperatures. The disadvantage is that the much lower flux of neutron sources means that considerably larger crystals (∼0.5 mm^3^ vs. 0.008 mm^3^) are essential and perdeuteration is desirable.^20^ These are likely to remain significant disadvantages of the method. Additionally, the development of time-resolved serial femtosecond crystallography (tr-SFX) at X-FELs may render both neutron and synchrotron studies of proteins obsolete.^21^

However, an area where nondestructive methods are essential is the study of cultural heritage objects^22^ and neutrons excel in this arena. The high penetration of neutrons means that bulk composition can be determined. Neutron radiography and tomography provide complementary approaches to the X-ray methods.^23^

While thermal neutrons are largely nondestructive, high energy (MeV) neutrons can cause damage in materials, both inorganic^24^ and organic.^25^ In nature, such neutrons are produced by high energy cosmic rays, mainly solar protons, interacting with the earth’s atmosphere and generating cascading showers of secondary particles. This is a particular risk for electronic components, even at the earth’s surface where the flux is low, as the number of components *e.g*. in automobiles, increases. The problem is even more important for avionics, because the atmospheric neutron flux is 300 times greater at typical aircraft altitudes than at sea level.

The need to be able test components in a reasonable time (a few hours) has led to the development of dedicated instruments that directly view the neutron target.^26^ The flux distribution produced by a spallation source matches that of the atmospheric neutrons, albeit ∼10^10^ times more intense. It is difficult to see where there may be a competing technology to neutrons.

Muons^2,27^ investigate the static and dynamic magnetic fields inside materials to reveal information about their properties.^28^ This can be used far beyond the obvious application to magnetic materials, from ionic motion in battery materials^29^ through liquid crystals^30^ to chemical reactions in solution.^31^ The commonly used abbreviation for the technique, μSR (Muon Spin Rotation/Relaxation/Resonance) was coined to highlight the analogy with NMR and ESR. In many contexts the applications of NMR and ESR are well-known. Muons implanted into samples address similar questions without necessarily requiring specific nuclei, radio frequency coils or applied magnetic fields, though these can all be useful in particular experiments.

**Figure 3:**
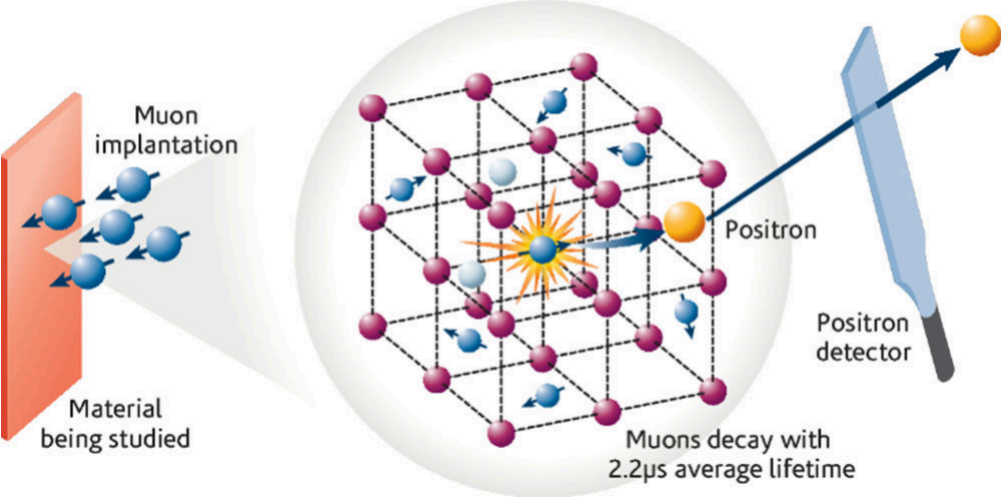
Muon implantation and decay. Reproduced with permission from ref [^32^]. Copyright 2016 ISIS Neutron and Muon Facility.

The short (2.2 μs) lifetime of the muon is advantageous in experiments because the resulting β particle is mostly likely to be emitted in the direction of the muon spin at the instant of decay, as illustrated in Figure 3.^32^ From the change in spin-polarization from muon implantation to muon decay, generally measured over millions of muon decays, the magnetic fields inside the sample can be inferred. Most muon experiments today use spin polarized beams of positive antimuons to measure the magnetic fields in bulk samples of 100 mg or more. These beams come from positive pions decaying at rest in the surface of the production target, so they are commonly called “surface muons”. The benefits of using these positive muons are that they are >95% spin-polarized, have a narrow momentum spread and generally stop within 0.1–1 mm in most materials. Beams coming from pions decaying in flight are called “decay muons”, allowing for higher and lower momenta and also negative muons. Decay muons can be used in pressure cells,^32^ in situations where a negative muon has a clearer interaction with the sample, or to use the X-rays emitted by nuclear capture of negative muons to determine the elements the sample contains, this has particular advantages for studies of cultural heritage objects.^33^ All of these techniques work with bulk samples but beams of positive muons can also be moderated to far lower energies, “slow muons” with keV rather than MeV energies, which are used to study thin film materials with thicknesses in the range 10–200 nm.^34^

Muons are used to study almost as many different scientific questions as neutrons, but in different ways.^2^ In magnetic and superconducting materials, the internal magnetic fields reflect the order parameters.^27,35^ Because muons implant at distinct crystallographic sites, but randomly over the bulk of a sample, a magnetically ordered state leads to specific muon spin precession frequencies whereas a superconducting state with a vortex lattice gives a distribution of fields over length scales sufficiently large for the muons to sample randomly. The random implantation over the bulk of the sample while sampling the properties local to the implantation site means that muons are volume sensitive, which is particularly useful in materials exhibiting multiple order parameters or multiple phases that do not occupy the full volume of the sample. In a battery material, muons investigate the rate of ionic motion by counting how frequently an ion moves close to them and flips the muon spin as the local fields change. In semiconductors and insulators, the positive muon generally captures an electron to form muonium, which behaves as an unstable light isotope of hydrogen. This enables studies of hydrogen defect behavior in semiconductors,^36^ chemical reactions of hydrogen by analogy and molecular dynamics.

Muons have proved particularly useful at examining weakly magnetic and disordered ground states, for determining the penetration depth of superconductors, impersonating hydrogen defects in semiconductors, and probing local charge motion in battery materials. These areas therefore make up the majority of contemporary muon spectroscopy research.

The, so-far, inexorable rise in computing power begs the question as to whether computational studies will render experimental investigations obsolete. This is a question that confronts much of experimental science, not just neutron scattering and muon spectroscopy. At present, computational studies need experimental validation. Whether the accuracy and reliability of such methods can be improved to the state that this is no longer necessary, is uncertain. Artificial intelligence methods rely on the availability of large data sets that are generated by experimental work. For example, the “solution” of the protein folding problem by AlphaFold^37^ used the Protein Data Bank^38^ for its training set. If the data sets are created by computational methods, this would be a circular argument. The old adage “garbage in, garbage out” is still true. The synergy between experiment and theory is likely to hold good for as long as science is done.

In summary, the present situation is that neutrons and muons provide complementary information to that provided by X-ray and other techniques. This is a consequence of the fundamental properties of the particles, so the need for neutron scattering and muon spectroscopy would seem unlikely to change in the next few decades.

## 3 What Might the Technology to Produce Neutron and Muon Beams Look Like in the 2050s?

### 3.1 The Neutron and Muon Ecosystem

Neutron scattering and muon spectroscopy techniques can address a very wide range of science, from chemistry to cultural heritage, but essentially all of the research carried out using them has to be done at large central facilities. There are no small scale laboratory based equivalents, so the central facilities are needed for all of the training, preparatory and underpinning work, as well as the most challenging experiments. This is quite different from, for example, synchrotron X-ray sources where laboratory X-ray equipment is widely available in universities and research institutes. All of the research that can be done using neutrons and muons is, therefore, intrinsically coupled to the available central facilities.

Neutrons can be provided by both reactor and accelerator-based sources,^39^ muons only by accelerators.^2^ The accelerators used to produce muons are also suitable for either neutron or nuclear/particle physics applications, so for both financial viability and scientific synergy reasons, muon sources are generally coupled to other uses of the same accelerator. It can take a decade or more to propose, plan, construct and bring a neutron/muon facility into operation, however, the operating lifetime may be 40 years, so the possible scientific opportunities for these techniques in the 2050s largely depend on the already existing facilities and projects.

For neutrons and muons to play their potential role requires not just a single top range source, but an ecosystem of sources that provides a range of capabilities matched to the experimental requirements, and sufficient capacity to accommodate a large and expert enough scientific community to make effective use of these capabilities in their research programmes. A leading facility will typically operate of order 20–40 different instruments simultaneously, and run 24/7 during operating periods. This enables on the order of 1000 different experiments per year for several thousand researchers.

**Figure 4:**
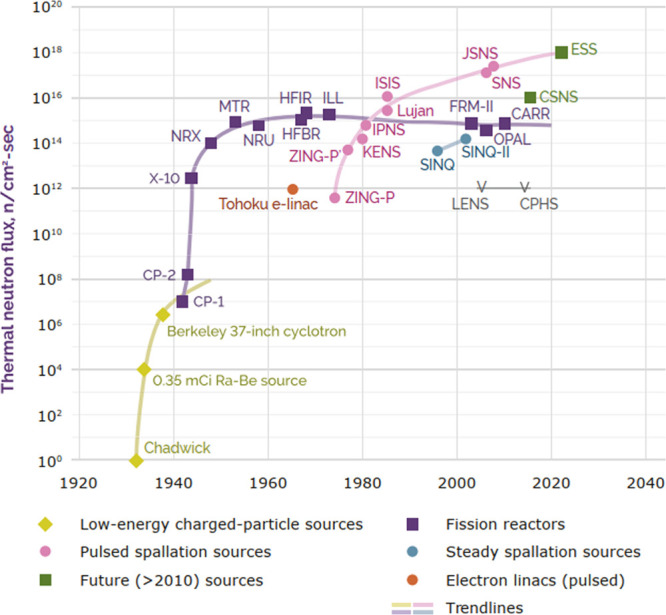
Evolution of effective neutron source fluxes as a function of calendar year, from the discovery of the neutron in 1932 to 2030. Reproduced from ref [^40^]. Copyright 2016 Dipartimento di Fisica - Università degli Studi di Milano.

In contrast to photon sources (e.g., synchrotrons, lasers), increases in neutron/muon source brightness have been modest over the last 50 years, see Figure 4. There are no “game changing” technologies on the horizon that will result in multiple orders of increase in source brightness by the 2050s, so the ESS running at 5 MW is likely to be the most intense source in 2050, (with the caveat that comparing pulsed and continuous sources is difficult, at best). However, there have been significant improvements in experimental capabilities mainly through developments in optics and detectors. Typically, a neutron instrument is “refreshed” about every 10 years: either replaced or upgraded. This usually results in a 10-fold improvement in performance through a combination of increased flux on sample by the use of neutron guides, improved detection efficiency (larger collection area, better detectors) and reduced background. Thus over the 50 years of neutron scattering, there have been major improvements in capability. In addition, developments in specialized sample synthesis, preparation and treatment, both off and on the beamline, have often been as transformative, particularly for chemistry applications.

Ultimately, facilities are judged on the quantity and quality of scientific output and the subsequent economic and societal impact. Two major factors that affect the conversion of experiments into output and impact are the reduced level of experience of the user community - who now typically employ a multitechnique and multidisciplinary approach to solve ever more complex research problems–as well as the challenge of increasing data volume and complexity. A particular advantage of neutrons is that the scattering cross-section is very simple (for most elements at most relevant energies) so an expected spectrum can be calculated very directly from a computer simulation.^41,42^ Indeed simulations, both classical^43^ and *ab initio*(44) molecular dynamics and density functional theory,^42,44−46^ are becoming a standard part of the data interpretation for many chemistry experiments. Machine learning is also beginning to be similarly applied.^47^

One other key factor on the time scale of the 2050s will be the approach to Net Zero. Neutron and muon facilities, even if operated using green electricity, have a significant carbon footprint. But what must also be considered is the broader benefit in carbon reduction contributed to by the research carried out–though methodologies for assessing this still need to be developed. However, as a simple example, a small improvement in the efficiency of an industrial chemical process enabled by the development or improvement of a catalyst could easily outweigh the carbon cost of running an entire facility for a year.^48^

### 3.2 Current Status and Future Development

#### Europe

Europe has historically had a larger number of neutron and muon facilities than other areas of the world. The Institut Laue Langevin (ILL) has been a leading neutron facility for five decades. It is the largest scale neutron facility in the world, with almost 40 instruments (including for fundamental and nuclear physics) and is supported by 14 member countries. The European Spallation Source (ESS), under construction in Sweden and supported by 13 countries, is scheduled to begin operation in 2026; the initial 15 instruments and 2 MW source power will provide capability beyond what currently exists, progressively opening new research opportunities.

The ILL reactor is currently planned to stop operation between 2030 and 2033; a decision will be made during 2024. A major program of maintenance work on key reactor components has recently been completed, as has the second phase of the “Endurance” instrument upgrade program. ILL can, therefore, continue to make a significant scientific contribution for the next decade, but it will not be operating in the 2050s. The reactor closure will have a major impact on the European ecosystem, being only partly compensated in the short term by ESS. However, by the 2050s it would be expected that ESS has reached its design power of 5 MW and full capacity of 35 instruments.

The large national facilities within Europe - ISIS in the UK, the Heinz Maier-Leibnitz Zentrum (MLZ) in Germany and the Swiss Spallation Neutron Source (SINQ)/Swiss Muon Source (SμS), also have a significant international user base. With 20–30 instruments in operation at each facility they provide more than half the capacity in the European ecosystem.

ISIS operates both neutron and muon facilities in an integrated way. The number of neutron instruments was significantly increased by the addition of a second target station, completed in 2009. The Endeavor program (2023–2030) will upgrade several instruments, both neutron and muon, and increase the number from 31 to 35. However, some of the basic ISIS infrastructure is now over 50 years old and future maintenance will become increasingly difficult. A new facility, ISIS-II,^49^ is in the early stages of conceptual design with operation potentially beginning after 2040.

Japan built a muon facility (RIKEN-RAL) at ISIS which started operation in 1995. After 2018, due to the muon facility at MLF coming into operation (see below), this facility was gradually transferred to ISIS.

MLZ is Europe’s most recent reactor based source. The FRM-II reactor has been undergoing refurbishment work in the past two years, but should restart operation in mid-2024 with instruments in the second guide hall operational. Further upgrades to the first guide hall are planned. This will increase the number of instruments from 27 to 33, but the potential exists for a further increase to 40. From a technical perspective FRM-II should be able to operate for at least another 20 years.

SINQ, an accelerator-based spallation source providing continuous rather than pulsed neutron beams, is unique in the world. The most recent major upgrade, completed in 2020, significantly improved the performance of the majority of the 14 instruments in operation. The option to construct a second guide hall for an additional seven instruments - increasing overall capacity by 50% for a fraction of the overall cost of SINQ - is currently being explored. A preliminary study will be completed by 2025, with construction possible toward the end of the decade. SμS utilizes the same accelerator as SINQ and so provides continuous muon beams which are very complementary to the pulsed beams at ISIS.

The current European ecosystem is completed by the smaller national facilities. The modernization program at the Budapest Neutron Centre (BNC) in Hungary, due to be finished in 2024, should enable the continued operation of the facility for a number of years while increasing the number of instruments from 12 to 15. Previous upgrades carried out at the Nuclear Physics Laboratory (NPL) in the Czech Republic have ensured that the facility can continue to operate until at least 2030. At the National Centre for Nuclear Research (MARIA) in Poland, refurbishments due to be completed by 2024 will make five additional instruments - transferred from the BER II reactor–available to users. The OYSTER upgrade program at the Reactor Institute Delft (RID) in The Netherlands will increase the number of instruments operated to seven with the option to add one more. However, it is unlikely that any of these facilities will still be in operation in 2050.

Several neutron “knowledge centers” have been created across Europe where sources have closed. These include the Jülich Centre for Neutron Science (JCNS, Germany – FRJ-2 reactor closed in 2006), the Laboratoire Léon Brillouin (LLB, France - Orphée reactor closed in 2019), the German Engineering Materials Science Centre (GEMS, Germany - FRG-1 reactor closed in 2010) and the Institute for Energy Technology (IFE, Norway - JEEP-II reactor closed in 2019). These centers support both their national and the wider European user communities through the operation of instruments at various operating facilities, in addition to their involvement in the construction of new instruments at ESS and the collaborative development of new neutron technologies.

In Russia, neutron research is supported by two national facilities: the IBR-2 pulsed reactor in operation at the Joint Institute for Nuclear Research (JINR) and the PIK reactor at the St Petersburg Nuclear Physics Institute, built in the 1970s but only now being commissioned for full power operation. There were plans to internationalize PIK, but given the current political situation this is unlikely in the foreseeable future.

#### Americas

The Oak Ridge National Laboratory (ORNL) hosts two major facilities: the High Flux Isotope Reactor (HFIR), the most powerful reactor-based source of neutrons in the country, and the Spallation Neutron Source (SNS). SNS is currently upgrading the accelerator power from 1.4 MW to 2.7 MW. 0.7 MW of the additional power is intended to be fed to a second target station (STS), which is in the detailed design phase, but the decision to fund/start construction has been delayed so it is unlikely to be operational before the mid-2030s. However, both SNS and STS should be operational well beyond 2050. There have been proposals for a muon source using the same accelerator, but this seems unlikely until after STS is completed.

HFIR has now been operating since 1965, so significant refurbishment is required. A proposal for a new reactor has not been taken forward. Replacement of the Be reflector, planned for 2028, will extend the operating lifetime and provide the opportunity for a major upgrade of the instruments. However, future replacement of the pressure vessel would be needed if HFIR is to operate into the 2050s, and this has not yet been decided.

A range of world-class capabilities are provided at the National Institute of Standards and Technology’s reactor-based source, the NIST Center for Neutron Research (NCNR). NCNR has not been operational since 2021 due to damage to a fuel element, but user operation is now expected to restart in 2026. The reactor has been operational since 1967. Recently there have been discussions around the requirements for a new reactor, and the business case is in preparation. The existing reactor would be unlikely to operate until 2050, but a new reactor could be operational on this time scale if a decision was made soon.

The accelerator-based Los Alamos Neutron Science Centre (LANSCE) is still operational but the main external user program for neutron scattering was stopped after SNS started operation. However, a small program continues on a limited number of instruments. The Missouri University Research Reactor (MURR) operates four neutron scattering instruments and is licensed until 2037, but seems unlikely to be still operating in the 2050s.

In Canada, the only major neutron source, the National Research Universal (NRU) reactor in Chalk River, was closed in 2018. New investment is being made at the small McMaster University reactor and new collaborations to provide access to neutron facilities in the US and other countries for Canadian users have commenced. There are ongoing discussions either about a future research reactor, or about a small compact source (see future facilities below), but these would need to make progress soon if they are to contribute by the 2050s. The TRIUMF accelerator center in Canada does not provide neutron beams (for scattering), but does provide capabilities for muon spectroscopy. Like SμS, these are continuous beams of both positive and negative muons.

Reactor-based neutron sources are currently under construction in Argentina (RA-10) and Brazil (Brazilian Multipurpose Reactor RMB). Both are being developed by the Argentinian company INVAP and are based on the OPAL design built for Australia, so potentially could have similar research capabilities. RA-10 is close to completion. Progress is strongly affected by the political situations in these countries, but they should be operational well before 2050.

#### Asia-Oceania

Japan hosts two major world-class facilities: the Japan Research Reactor (JRR-3M) and the Materials and Life Science Facility (MLF) at the Japan Proton Accelerator Research Complex (J-PARC). MLF also hosts instruments for muon spectroscopy and fundamental physics. The MLF target station now has very limited scope left to expand the number of instruments (upgrades are possible), so early discussion has started about the building of a second target station with both neutron instruments and an innovative/ambitious muon source. This could be operational by the 2050s, but no formal proposal or decision has yet been made. JRR-3 M returned to operation in 2021, having been shut down following the 2011 earthquake. Given that the core had been replaced in 1990, the reactor should technically be able to provide capabilities until 2050.

The strategic importance of neutrons has been established in China and neutron research, supported by significant investment, is now increasing accordingly. Capacity is provided by three main facilities: the China Advanced Research Reactor (CARR), the China Mianyang Research Reactor (CMRR), and the China Spallation Neutron Source (CSNS). Instrumentation at CARR and CMRR is limited and developing only slowly. However, CSNS, which started operation in 2019 with only three instruments, is now rapidly expanding with over ten more in construction/design, including one for muon spectroscopy. A second target station, for which provision was made in the original facility design, may well be constructed within a few years.

CiADS is a combination of a high power proton accelerator and a subcritical reactor, under construction in China, intended to develop ADS technologies for nuclear waste transmutation. A muon facility has been proposed as an adjunct to this facility, but the possible timeline for this is not clear.

Since 1995 South Korea has operated a multipurpose research reactor, HANARO, equipped with 15 instruments for neutron science. However, the reactor has only operated intermittently for some years and, since the construction of a spacious guide hall, investment in instrumentation has been limited. The accelerator complex RAON, which is under construction, is mainly focused on nuclear physics but a facility for muon spectroscopy has been proposed and could be available from the 2030s.

Neutron research in Oceania takes place at the Open Pool Australian Lightwater (OPAL) reactor, a large national facility operational since 2007. OPAL is one of the few research reactors for neutron science that could significantly increase its instrumentation beyond what is currently required by the country’s user community, through the construction of a second guide hall. This is probably the most cost-effective opportunity worldwide, but would require international partners and distance makes the logistics more difficult.

There are many other research reactors around the world,^9^ in addition to those mentioned above, which offer limited neutron scattering capability. However, equipment tends to be old and investment is limited, so major development in the next 20 years is unlikely. The exception might be if India decides to build a new reactor to replace the Dhruva reactor at the Bhabha Atomic Research Centre (BARC), which has now been operating for nearly 40 years.

**Figure 5:**
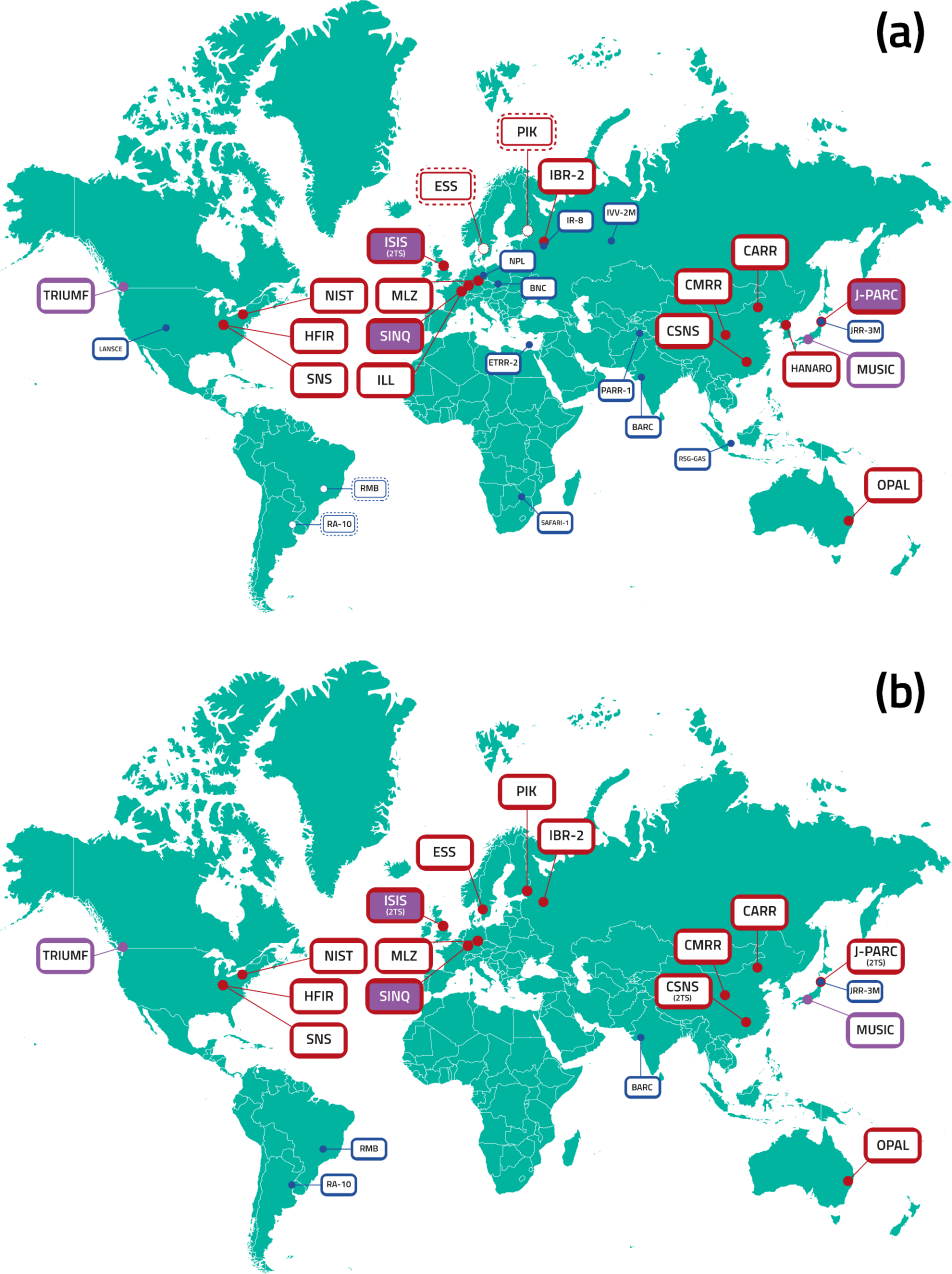
Neutron and muon facilities worldwide: (a) those that are operating in 2024 and (b) those likely to be operating in the 2050s. Larger facilities are shown in red, smaller ones in blue. Magenta are muon facilities. Combined facilities that have both neutron and muon capabilities are those with a red outline and a magenta fill. Facilities with two neutron target stations are indicated by (2TS). Dashed lines indicate a facility that is under construction. This figure was adapted by Dr Stephanie Richardson with permission from ref [^50^]. Copyright 2022 BrightnESS^2^ project.

Figure 5 shows the neutron and muon facilities currently operating (Figure 5a) and those that are likely to be operating in the 2050s (Figure 5b).

### 3.3 Future Facilities

#### Neutrons

It seems unlikely that a new high-flux reactor of similar or higher specification to ILL or HFIR will be built for neutron scattering. Accelerator-based spallation sources can now provide similar or greater neutron science capability at an equivalent capital cost. The primary rationale for building a high-flux reactor would then be for the production of specific neutron-rich isotopes for a range of applications, including cancer therapy; neutron science would be secondary. Medium-flux reactors - similar to OPAL - can be built on a commercial basis (as in Argentina and Brazil), providing capacity and capability locally on a relatively quick time scale and at a cost that is viable for many individual countries, though again isotope production would be an important factor. Renewed global interest in nuclear energy may result in several such projects developing before the 2050s.

**Figure 6:**
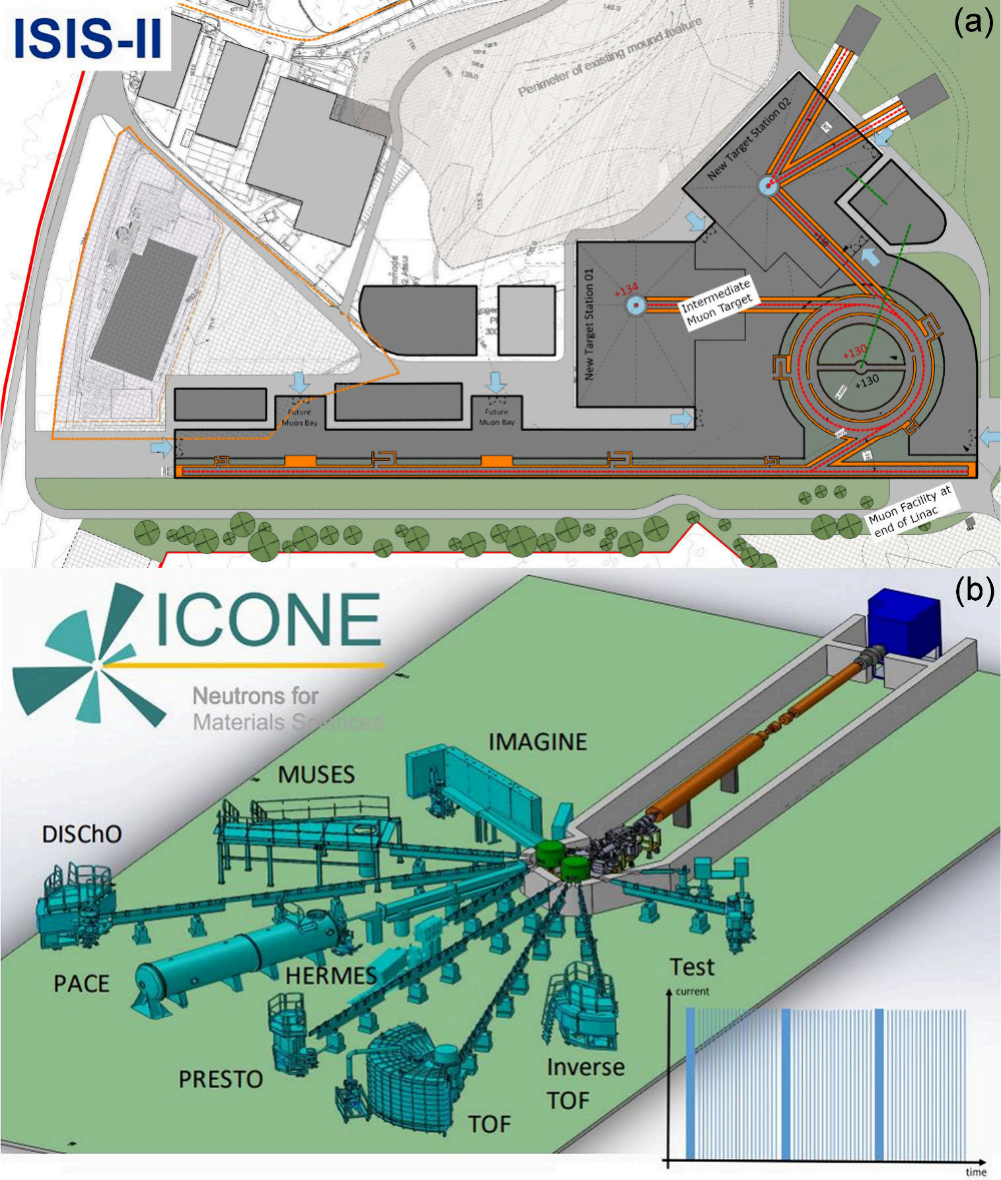
(a) Schematic of a state-of-the-art neutron facility in the 2050s using a superconducting linac and running two target stations. Possible positions for muon targets at the end of the linac or before a neutron target are shown. Based on a possible design for ISIS-II (UK)^48^ and (b) schematic of a HiCANS-type spallation source, ICONE (France).^51^ Reproduced with permission of the ISIS Neutron and Muon source and under a Creative Commons Attribution License 4.0 (CC-BY) from ref [^52^]. Copyright 2023 edpsciences.

Accelerator-based spallation sources offer a route to high performance but, because of the high accelerator energy required, have a high “entry price” in terms of construction and operating costs. With one such source having been built in each of the major regions since 2000, the only other potential such source under consideration is ISIS-II. China could possibly build another accelerator source, in addition to a second target station for CSNS, but the current focus there is on electron synchrotrons. Figure 6a illustrates what a state-of-the-art large scale neutron and muon facility might look like in the 2050s. Given the long lead time for such facilities, it is likely to be similar to current state-of-the art facilities, SNS, J-PARC and ESS, in that it will use a superconducting linac for proton acceleration.

As an alternative to medium flux reactors for national neutron sources there is now increased interest in High Current Accelerator-driven Neutron Sources (HiCANS).^51^ These are accelerator-driven sources that produce neutrons through lower energy nuclear reactions rather than high energy spallation. Though low energy sources, often known as Compact Accelerator-driven Neutron Sources (CANS), have existed for many years, the capabilities were relatively limited. Developments in accelerators, target design, moderators and neutron technologies now offer the possibility to deliver significantly enhanced neutron science performance. Due to their considerable flexibility - in terms of cost, capacity and capability - HiCANS could play an important role in sustaining the international neutron science ecosystem. The most advanced projects of this type are ICONE^52,53^ in France and the High Brilliance Source (HBS)^54^ in Germany. However, a first HiCANS facility now needs to be built to demonstrate the potential of this approach. Figure 6b illustrates a possible design for a small scale spallation source neutron facility.

Laser-based techniques for neutron generation have been demonstrated using either laser-induced fusion or “plasma wakefield” acceleration. However, the possible extension to a neutron facility with multiple instruments has not been properly explored.^50^

#### Muons

Muons are usually produced by a high energy proton beam incident on a carbon target, which allows most of the protons to pass through it for another use. Looking at the stated future plans of the existing muon facilities, it is likely that at least some muon facilities will still be using this approach in the 2050s while making significant improvements in source performance.

The threshold energy for producing muons using a proton beam incident on a stationary target is E_p_ > 280 MeV. This produces single pions that decay into muons and production peaks between 500 < E_p_ < 1000 MeV. Higher energy beams with E_p_ > 600 MeV can produce two pions, with the production rate saturating around 1.5 GeV. The present muon sources either use cyclotrons to produce continuous muon beams or synchrotrons to produce pulsed muon beams. Other accelerator technologies could equally well or better produce such proton beams, such as nonscaling fixed-field alternating gradient (ns-FFAG),^55^ linacs, and laser wakefield accelerators.^56−59^ Choices between such options may well be driven by how well these technologies meet the requirements of whatever sits alongside the muon facility. In the future, these might include accelerator-driven nuclear reactors, as is being developed at CiADS,^60^ or accelerators for proton therapy.

More exotic methods for producing muons using an intense GeV electron beam or using ion accelerators have also been proposed. Muon production, capture, and transport are highly active fields of study in the particle physics community, both for high energies leading to a muon collider and for lower energies leading to precision measurements. These developments may lead to improved muon sources for muon spectroscopy.

One of the areas in which particle physics design studies have proposed improvements is production target technology. Present muon sources use graphite targets, since it is readily available, operates well at high temperatures and has a high muon production cross-section. At the MW muon sources it has proven necessary to rotate a graphite wheel to spread the heat load but studies for higher power muon sources suggest that other materials will be needed or could at least give a higher muon yield. This could use a liquid metal jet or a higher atomic number metal e.g. tungsten. Across the periodic table there is a wide variety of elements with high production cross sections, so target materials will likely be chosen based on whether the beam needs to be transported to another downstream target, or for engineering reasons.

Other areas that are likely to see significant developments are improving the “quality” of the muon beam after it has been produced and adopting more advanced detector technologies. The former will be a continuation of work that removed unwanted particles from the muon beam, rotated the muon spins in flight and split up separate pulses of muons to different beamlines. The latter should finally move beyond a solid scintillator coupled to a light sensor akin to a photomultiplier tube. At pulsed muon sources, reducing the time-width of the pulses below the pion lifetime will open up a range of studies of larger magnetic fields inside materials that have hitherto been the preserve of continuous muon sources measuring individual muons at a time.^61−63^ At continuous sources, tracking muons into a sample and decay positrons from the sample should overcome the single muon at a time rate limitation of today’s instruments. Such tracking detectors could widely allow multiple volumes within a sample to be mapped, a sort of muon microscopy. At typical muon energies and sample densities the range of muon stopping positions in a sample is around (250 μm)^3^. These length scales are relatively large compared to other microscopy and radiography techniques so this is likely to be an extension of muon spectroscopy rather than a replacement of other techniques.

To reach into far smaller volumes within materials using muons needs them to be traveling far more slowly than when they leave the production target. This is sometimes referred to as muon cooling or slow muons. The Low Energy Muon (LEM) beamline at PSI^64^ has pioneered this approach, cooling a surface muon beam from 4 MeV to eV in a moderator, then reaccelerating it to keV energies that can be implanted between 10 and 200 nm into the surface of a material, all while retaining the spin polarization. Other ways of producing similar slow muon beams have been prototyped at RIKEN-RAL and J-PARC, using laser ionization of muonium^65^ and in the muCool^66^ experiment at PSI, using cryogenic He gas moderation. All of these approaches have to confront the inefficiency inherent to cooling muons. For the LEM beamline, around 10^–4^ of the initial muon beam reaches the sample. A similar efficiency has been reported for the laser ionization of muonium but the muCool approach is around 10^–3^ efficient and also reduces the beam spot size. An extension of muon cooling and beam compression is reaccelerating the beam, somewhat like an electron microscope, which could reduce the extent of the beams from mm to 10 μm, at energies between 200 keV and 1 MeV.

Another aspect is the production and understanding of muon spectroscopy data. Until recently the data sets generated by instruments have generally been quite small, <100 MB day^–1^. However, data are now being collected as events and instruments in the near future will generate >10 GB day^–1^, still well within present computing capabilities. Significant progress has also been made recently in understanding the muon stopping sites using DFT calculations.^67^ This has furthered the understanding of how muons perturb their environment in some materials and enabled the explicit calculation of relaxation functions in simpler materials. This is likely to be extended to more complex materials in the near future. Another interesting direction for muon data analysis is the application of machine learning approaches. This has been explored using principal component analysis and found to be effective in identifying the form of even very small data sets and tracking phase transitions.^68^ Future applications of similar tools may change how muon data are collected, allowing for better use of limited beamtime and perhaps even more automated experiments.

Figure 6a illustrates a possible configuration for how a muon facility could be part of a neutron spallation source in the 2050s. Table 3 compares present and future muon sources of different types, showing how their strengths and weaknesses are likely to remain complementary in the future.

**Table 3:** Comparison between Present and Future Muon Sources^a^

Property	Present continuous	Future continuous	Present pulsed	Future pulsed
Rate [TD] (MHz)	0.006	0.1	0.04	1
Rate [TI] (MHz)	1	100	0.04	1
Time range (ns)	0.1–10000	0.1–10000	100–30000	10–30000
Resolution (TD) (ps)	100	100	100,000	10,000

a Properties of continuous and pulsed muon sources, both today and in the future after currently planned improvements have been completed. Muon measurements either record the muon spin polarization as a function of time after implantation (time-differential, TD) or else measure a time average muon spin polarization (time integral, TI). Adapted from ref [^2^] with permission of Oxford University Press through PLSclear.

## 4 What Will Be the Applications in the 2050s?

### 4.1 Neutrons

This question is the hardest to answer. Neither the high temperature superconductors nor fullerenes were predicted in the 1980s, yet they are now major areas of research by both academia and industry. There will undoubtedly be other paradigm changing discoveries in the next quarter century. If these contain light elements (and in particular hydrogen), these will require investigation by neutron scattering and muon spectroscopy as there are very few other techniques that can investigate the structure so precisely. A case in point is the current activity in the search for superconducting “superhydrides” where the number of hydride ions exceeds normal valence rules *e.g*. CaH_2_ vs CaH_6_.^69^ When (if) such materials become available in large enough quantities (10s of mg), neutron scattering to determine the hydride and magnetic structures and muon spectroscopy to investigate the local magnetism will be essential.

A trend that is apparent now, is that experiments are becoming more complex. Whereas ten years ago, a single variable *e.g*. temperature, pressure or composition, was investigated, today there is demand for low temperature (<4.2 K), high pressure (MPa - GPa) and high magnetic field (>5 T) simultaneously. There is also demand for concurrent measurements. While some inelastic neutron scattering instruments have long had a simultaneous neutron diffraction capability, (in fact VESUVIO^70^ at ISIS has five neutron techniques that are measured concurrently) the drive is to toward complementary non-neutron techniques. Diffraction and conductivity^71^ or dielectric measurements, Raman spectroscopy with inelastic^72,73^ or elastic neutron scattering are now available or planned at several facilities. The implementation of UV–vis spectroscopy on a neutron beamline would be straightforward. Differential scanning calorimetry (DSC)^74^ or electrochemical impedance spectroscopy (EIS)^75^ with QENS is already available at ISIS. Perhaps the most useful spectroscopic technique would be infrared spectroscopy. This is used with neutron reflectometry, as the relatively open geometry of reflectometers allows the spectrometer to be closely positioned to the sample. But in general, this has been hard to implement because infrared light sources are weak and mid-infrared fiber optics are expensive and have limited long wavelength transmission. However, the advent of quantum cascade lasers (QCL) offers broad band coverage and, when the cost decreases, are likely to be the method of choice. Neutron imaging with simultaneous spatially resolved diffraction measurements are under development on IMAT^76^ at ISIS. NEXT^77^ at the ILL was designed to carry-out simultaneous neutron and X-ray tomography. Simultaneous small-angle X-ray (SAXS) and neutron scattering (SANS) has been implemented on D22^78^ at the ILL and provides a complementary contrast mechanism.

One area that will likely see significant growth is that of the use of polarized neutrons. To date, the limiting factor has been the low fluxes that result from the polarization process. Neutron diffractometers have used polarization analysis to separate magnetic, coherent and isotope incoherent and spin incoherent cross section components, as exemplified by D7 at the ILL.^79^ Neutron imaging of magnetic fields using polarized neutrons has been demonstrated.^80,81^ A recent development has been the use of polarization analysis for QENS.^82^ The technique is still in its infancy, but it is already clear that it will revolutionize the field, particularly when instruments with polarization analysis as an integral component become available, such as the proposed spectrometer SHERPA^83^ at ISIS.

Neutron scattering instruments are generally spacious (10s to 100s cm^3^ available at the sample position) so adding multiple capabilities is feasible.

One area that has seen huge growth over the last 50 years is the amount of data generated per instrument. This is largely driven by the trend to maximize the detector coverage to at least partially compensate for the relatively low flux of neutron sources. State-of-the-art instruments are approaching 4π sr coverage, thus the next upgrade to TOSCA at ISIS will result in an increase from 1 to 6.12 sr and including detector and filter improvements, an overall count rate increase of 11.2.^84^ However, this is still well within current technologies. Count rates at the ESS instruments will be significantly greater than the current generation of instruments but are still manageable.

The much bigger challenge will be data analysis. At all large scale facilities (both neutron and synchrotron), the rate limiting step to generating output is data analysis not data acquisition. The rise in computing power has meant that more sophisticated analysis is possible but increasingly it is the staff at central facilities that are expected to carry-out the analysis and there is a finite (and relatively small) number of these. How to “democratize” data analysis is a problem that all central facilities are wrestling with and to which there is no easy solution.

### 4.2 Muons

Looking back at the history of muon spectroscopy, its applications seem to form a series of punctuated equilibria. Applications in magnetism have remained steady whereas superconductivity saw a huge increase in activity following the discovery of high-Tc cuprates, which has only gradually subsided even as the superconducting materials studied have changed. The growth in superconductivity research coincided with a decrease in studies of muon diffusion in metals, as researchers changed topic and brought in new groups from a newly invigorated field. A similar change happened more recently as the muon methods used to study ion diffusion in solids improved, making studies of battery materials routine just as they became far more widely studied. The crystal ball showing similar changes in the scientific topics studied is likely to be rather cloudy, but one can draw some conclusions based on technical changes.

Clear indicators are where particular facilities have specific capabilities that are highly oversubscribed, which could be replicated there and elsewhere to meet this demand. The most obvious is the demand for low-energy muons for surface and interface studies. Many of the research areas in which muons are particularly effective in the bulk are also extensively studied as thin films. The way the depth probed can be controlled by varying the incoming muon energy makes them very powerful for studying layered materials. Doing pump–probe studies with muons like those already done to study charge carrier concentrations is likely to become more widespread as the availability of these measurements increases.

There are also some areas that seem ready to develop on the back of the increasing muon flux in existing facility plans. First among these is radio frequency μSR, which uses pulse sequences to gain greater control and precision over how the muon interacts with its environment. Most muon spin spectroscopy experiments are analogous to the earliest types of NMR measurements. NMR has developed enormously as more sophisticated pulse sequences have been applied. This is considerably more challenging for muons because of their short lifetime, but with higher muon fluxes comes the opportunity to spend longer manipulating the muon spins with more complex pulse sequences.^2^ Using muons for microscopy and tomography experiments has also been proposed and work on aspects of these developments is ongoing.^85,86^ Finally, just as neutron beams have become an important tool to understand single event upsets in semiconductor devices as a mimic of cosmic rays, so it is quite possible that muon beams will be necessary in future,^87^ both as semiconductor feature sizes decrease and also as quantum computing develops.

## 5 Conclusions

With the decades-long lead time required for major infrastructure projects, such as neutron and muon facilities, the hardware is likely to be similar to that used today. In particular, there is unlikely to be the orders of magnitude increase in incident flux that has been seen by synchrotrons over the last few decades. Gain factors in the range of a few tens (at best) of that of existing facilities are more likely. These will be amplified, to a degree, by continuing improvements in neutron guides, detector coverage and detector technology. The big difference will be how they are run: AI is already playing a role at the major facilities and this is clearly the direction of travel. Given that the cost is significant, not all of the potential new projects and facilities outlined will be realized and those that are will be highly international, as shown by the ESS.

However, many of the potential facility closures are almost certain. The USA “created” neutron scattering in the 1950s but the balance shifted decisively toward Europe with the advent of the ILL in 1975 and ISIS a decade later. With SNS (USA) and J-PARC (Japan) starting in the 2010s, the playing field leveled. However, in the 2050s, the neutron landscape in Europe will be much sparser; the reduction in capacity resulting from the closure of the ILL and most of the small reactors in the 2030s will only be partially compensated by ISIS-II and the ESS. The SNS is likely to be the only major neutron facility in north America and even with a second target station, there will be reduced capacity. In Asia, both CSNS (China) and J-PARC are likely to have second target stations and this represents the only geographical area where there will be increased capacity. Overall, the neutron/muon ecosystem in the 2050s will therefore have less capacity than now, but greater capability because of the somewhat higher power sources and continuing advances in neutron optics and detectors.

For muons, the situation in the 2050s is rosier. All of the existing facilities (or their successors *e.g*. ISIS-II), will be operational and there may well be additional capacity including muon beamlines at SNS and at CiADS (China).

In order to play an appropriate (and necessary) role in complex and multidisciplinary science, much of which will be focused on continuing global challenges, the facilities will need to be operated very efficiently. A key aspect will be to much more effectively exploit the data produced, either through databases and machine learning or tighter integration with computer simulation and modeling.

One of the key attributes of neutron scattering and muon spectroscopy is that they are nondestructive; so samples such as biological materials, cultural heritage artifacts or electronic devices can be examined without destroying or perturbing them. More generally, these are all areas of growth and offer possibilities for real time studies. There can be little doubt that neutron scattering and muon spectroscopy will both still be needed in the 2050s and beyond.
